# Appropriate use of meaningful within‐patient change (MWPC) thresholds in Alzheimer's disease

**DOI:** 10.1002/alz.14436

**Published:** 2024-12-18

**Authors:** Claire J. Lansdall, Jeffrey L. Cummings, Jeffrey Scott Andrews

**Affiliations:** ^1^ Product Development Patient‐Centered Outcomes Research, F. Hoffmann‐La Roche Ltd Basel Switzerland; ^2^ Chambers‐Grundy Center for Transformative Neuroscience University of Nevada, Las Vegas (UNLV) Las Vegas Nevada USA; ^3^ Global Evidence & Outcomes Takeda Pharmaceutical Company Limited Cambridge Massachusetts USA

1

Determining whether disease‐modifying treatments (DMTs) in early Alzheimer's disease (AD) provide clinically meaningful benefits to people living with AD is critical and has triggered much debate in the field.^1^, ^2^, ^3^, ^4^ AD is a slowly progressive, ultimately fatal, neurodegenerative disease, characterized by progressive loss in cognitive ability and daily function.^5^ Current DMTs aim to *slow* disease progression, an important treatment‐related outcome for people with, or at risk for, AD and their care partners.^6^

A widely adopted approach to evaluate the clinical relevance of a treatment benefit is to compare the proportion of patients who experience a meaningful improvement or deterioration in their symptoms and/or overall condition over the course of a clinical trial. In progressive diseases where emerging DMTs aim to slow the rate of progression in symptoms and underlying disease (as opposed to the temporary improvements provided by symptomatic therapies), evaluating meaningful deterioration is arguably more appropriate. To conduct such analyses in AD, estimates of meaningful score change on the Clinical Dementia Rating–Sum of Boxes (CDR‐SB) are needed to reflect clinically relevant deterioration in an individual patient's cognition and daily function. Lansdall et al.^7^ used established methods to generate *meaningful within‐patient change* (MWPC) thresholds to define meaningful deterioration on the CDR‐SB, building on existing estimates.^8^, ^9^ This letter aims to provide clarification on the appropriate application of MWPC thresholds to evaluate clinical trial data.

Thresholds to define meaningful individual‐level change on a scale are commonly used in neurology trials, both to identify responders and progressors. Examples include confirmed disability progression on the clinician‐administered Expanded Disability Status Scale in multiple sclerosis or meaningful reduction of neuropathic pain on a pain intensity numeric rating scale.^10^, ^11^ Figure 5 of the FDA Aricept (donepezil) label, a symptomatic treatment for AD, also includes an example of how to apply MWPC thresholds to clinical trial data, using 4 and 7 points improvement on the Alzheimer's Disease Assessment Scale–Cognitive Subscale (ADAS‐Cog) to report the proportion of responders across treatment and placebo arms. Although the intended use of these thresholds is to define and assess MWPC, recent publications in AD have instead applied MWPC thresholds to evaluate whether the magnitude of observed between‐group differences in mean change‐from‐baseline (commonly the primary endpoint in AD trials) is clinically meaningful.^3^, ^4^ Trigg et al., highlight why MWPC thresholds cannot be applied directly to evaluate the meaningfulness of between‐group differences,^12^ and doing so sets an inappropriate bar for emerging DMTs in AD. For example, considering that the average observed placebo decline over 18 months in recent clinical trials of early AD of around 1.5–2.5 points on the CDR‐SB^13^, ^14^ is approximately equal to the proposed range of MWPC thresholds, a meaningful effect could be reached only if the treatment group remained stable or a large proportion of patients achieved a marked improvement from baseline. This expectation that treatments can recover what is already lost due to AD is unrealistic for emerging DMTs aiming to slow disease progression.

An appropriate application of MWPC thresholds would be to identify individual patients who may have changed or progressed to a *meaningful* degree to inform “progressor” analyses of trial data, which compare, for example, (a) the likelihood of meaningful progression (i.e., deterioration) for treatment versus placebo groups (i.e., hazard ratio analyses, Figure 1A), and/or (b) the proportion of meaningful progressors across treatment arms via progressor analyses and/or annotation of empirical cumulative distribution function (eCDF) plots (Figure 1B). Examples of similar analyses have been presented at recent conferences.^15^, ^16^ In the context of a progressive neurodegenerative disease, individuals will deteriorate to varying degrees over the course of a clinical trial. Acknowledging that not all patients will benefit from treatment and that those who benefit will do so to varying degrees, individual‐level analyses leveraging MWPC thresholds to demonstrate that fewer patients meaningfully progress may offer a more suitable approach to evaluate the meaningfulness of a treatment effect.

**FIGURE 1:**
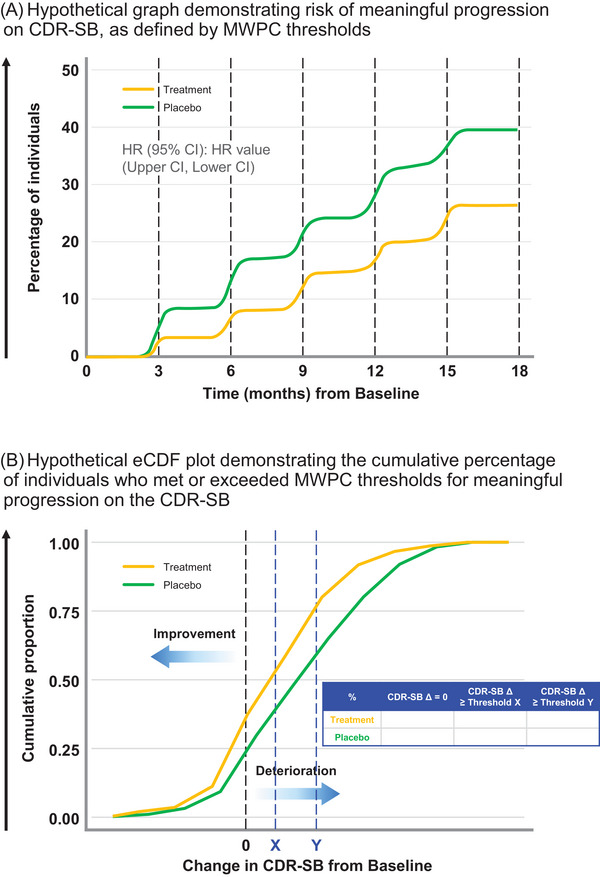
Hypothetical examples of analyses and outputs using MWPC thresholds to define meaningful within‐patient progression on the CDR‐SB. These outputs are for illustrative purposes only and reflect hypothetical trial data. (A) A hypothetical hazard ratio graph displaying the risk of meaningful within‐patient progression by treatment arm. Here, meaningful progression can be defined by MWPC thresholds that are considered appropriate for the enrolled trial population (i.e., it may be appropriate to select a 1‐point threshold for MCI and a 2‐point threshold for mild AD patients, based on existing estimates in early AD^7^, ^8^, ^9^). (B) A hypothetical eCDF plot, reporting the proportion of patients who meet or exceed established MWPC thresholds (denoted by Threshold X, Threshold Y) by the treatment arm in the table insert. eCDF plots can be useful graphical outputs to display the cumulative proportion of patients who experience a given score change on the CDR‐SB from baseline to the primary analysis timepoint. The curve for an effective treatment should be shifted to the left, indicating that individuals receiving treatment are less likely to experience greater levels of progression on the CDR‐SB. Graphs can be annotated with a range of MWPC thresholds that are considered appropriate for the enrolled trial population and the proportion of patients in each arm whose change‐from‐baseline meets or exceeds these thresholds can be reported as indicated in the table insert. AD, Alzheimer's disease; CDR‐SB, Clinical Dementia Rating—Sum of Boxes; eCDF, empirical cumulative distribution function; MCI, mild cognitive impairment; MWPC, meaningful within‐patient change.

A lack of clear and consistent terminology may have contributed to the confusion regarding the application of existing thresholds, with terms like minimal clinically important difference (MCID) often being used interchangeably to refer to both within‐patient change and between‐group differences. For example, Andrews et al.^8^ used methods to establish within‐patient change thresholds for the CDR‐SB, but use of the term MCID may have led to recent publications citing this work to evaluate the meaningfulness of between‐group differences.^3^, ^4^ For this reason, we use MWPC to clarify the intended use of these thresholds and align with recent publications.^1^, ^10^, ^12^ When used appropriately for individual‐level analyses, these thresholds provide an opportunity to build on typical primary endpoint analyses that evaluate between‐group differences in change‐from‐baseline and add to the totality of evidence for consideration when evaluating the clinical meaningfulness of a given treatment.^1^, ^2^, ^6^ Such analyses, including event‐based, dichotomous endpoints, may offer particular value for certain stakeholders and represent one important approach to evaluating the clinical meaningfulness of trial data.

## CONFLICT OF INTEREST STATEMENT

Claire J. Lansdall (C.J.L.) is an employee of F. Hoffmann‐La Roche Ltd and owns stock options in F. Hoffmann‐La Roche Ltd. Jeffrey L. Cummings (J.L.C.) has provided consultation to Acadia, Actinogen, Acumen, AlphaCognition, ALZpath, Aprinoia, AriBio, Artery, Biogen, Biohaven, BioVie, BioXcel, Bristol‐Myers Squibb, Cassava, Cerecin, Diadem, Eisai, Global Alzheimer's Platform (GAP) Foundation, GemVax, Janssen, Jocasta, Karuna, Lighthouse, Lilly, Lundbeck, EQT Life Sciences (formerly LSP), Mangrove Therapeutics, Merck, NervGen, New Amsterdam, Novo Nordisk, Oligomerix, Ono, Optoceutics, Otsuka, Oxford Brain Diagnostics, Prothena, reMYND, Roche, Sage Therapeutics, Signant Health, Simcere, Sinaptica, Suven, TrueBinding, Vaxxinity, and Wren pharmaceutical, assessment, and investment companies. J.L.C. owns the copyright of the Neuropsychiatric Inventory. J.L.C. has stocks/options in Artery, Vaxxinity, Behrens, Alzheon, MedAvante‐Prophase, and Acumen. J.L.C. has received the following grants: National Institute of General Medical Sciences (NIGMS) grant P20GM109025; National Institute of Neurological Disorders and Stroke (NINDS) grant U01NS093334; National Institute on Aging (NIA) grant R01AG053798; NIA grant P30AG072959; NIA grant R35AG71476; NIA R25 AG083721‐01; Alzheimer's Disease Drug Discovery Foundation (ADDF); Ted and Maria Quirk Endowment; Joy Chambers‐Grundy Endowment. Jeffrey Scott Andrews (J.S.A.) is an employee of Takeda Pharmaceutical Company Limited and a minor shareholder of Takeda Pharmaceutical Company Limited. Author disclosures are available in the Supporting Information.

## Supporting information



Supporting Information
